# Arising from the Ashes? Environmental Health in Detroit

**DOI:** 10.1289/ehp.122-A324

**Published:** 2014-12-01

**Authors:** Tim Lougheed

**Affiliations:** Tim Lougheed has worked as a freelance writer in Ottawa, Canada, since 1991. A past president of the Canadian Science Writers’ Association, he covers a broad range of topics in science, technology, medicine, and education.

For most kids growing up in Windsor, Ontario, the intensely industrialized landscape of Detroit is easily seen from the Canadian side of the river but almost never visited, says writer Tim Lougheed. As one of those kids, Tim welcomed an invitation from the Institute for Journalism and Natural Resources to take an intensive tour of this challenging and controversial urban landscape.

Many established industrial centers across the United States endured a rough introduction to the global economy during the second half of the twentieth century, but few matched the roller coaster ride of Detroit, Michigan. The city emerged from World War II as the triumphant forge for what President Franklin Delano Roosevelt dubbed the “great arsenal of democracy.”^1^ Factories that had once churned out passenger cars and their components now supplied the U.S. military with all manner of vehicles, weapons, and other equipment. By the 1950s the city’s consumer economy had been restored, along with Detroit’s status as the epicenter of North American automobile production and a host of thriving smokestack industries.

**Figure:**
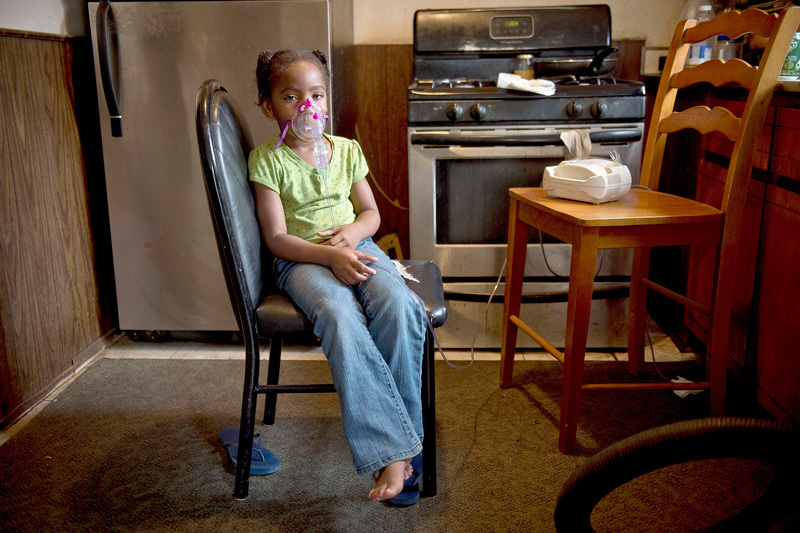
La’Miya, age 5, uses a nebulizer at her home in River Rouge, a suburb of Detroit. Although some asthma indicators have improved in recent years, Detroit’s hospitalization rate for the disease is three times the state average.^52^ © Ami Vitale/Panos Pictures

But already an exodus had begun. The urban population peaked at more than 1.8 million in 1950.^2^ Today’s population sits at just over a third of that number,^3^ a statistic that speaks to how far this urban landscape has been reshaped during the past few decades. Race riots, economic devastation, political corruption, municipal bankruptcy—Detroit has withstood some staggering socioeconomic blows, mirrored by severe environmental health concerns.^4^^,^^5^^,^^6^^,^^7^^,^^8^

Without visiting Detroit, it is easy to imagine a ruined metropolis, but even the most cursory inspection offers evidence of a remarkable resilience. Environmental and public health problems can still be readily found, but so too can testaments to a desire to move past this legacy and create something new. In the words of the city’s official motto, *Speramus meliora; resurget cineribus*: We hope for better things; it will arise from the ashes.

## Perception and Pollution

Those words hang on the wall of the Delray Neighborhood House, a small community center tucked into a crowded industrial setting southwest of Detroit’s downtown core. Next to the motto are framed portraits of a proud past, including local landmarks, elegant ferryboats plying the riverfront, and epic images of the Detroit International Exposition Fair of 1889, which vaulted Delray into the cutting edge of electrification and modern plumbing as befitted one of America’s most industrialized communities. Annexed by Detroit early in the twentieth century, city planners repeatedly drafted zoning strategies that intensified the area’s industrial character.^9^

By the 1960s, Highway I-75 had cut a swath through Delray; residents are preparing for further upheaval over the next few years, with the construction of a massive plaza to accommodate the road system for a new bridge to Canada. The city’s 2012 land use map shows a large amount of land designated M4—intensive industrial—in many cases abutting blocks designated R, for residential.^10^^,^^11^ Delray is among several parts of the city where declining population and rising poverty and unemployment meet the Michigan State Housing Development Authority’s definition of “distressed.”^12^

This label comes as no surprise to Rhonda Anderson, who grew up just north of Delray and has become an environmental justice organizer for the Sierra Club’s Detroit branch. She knows Delray Neighborhood House as a place for bringing residents together to discuss the environmental condition of their community, as well as the scene of meetings—sometimes contentious—with representatives from government and industry, whom the residents deem responsible for that condition.

**Figure:**
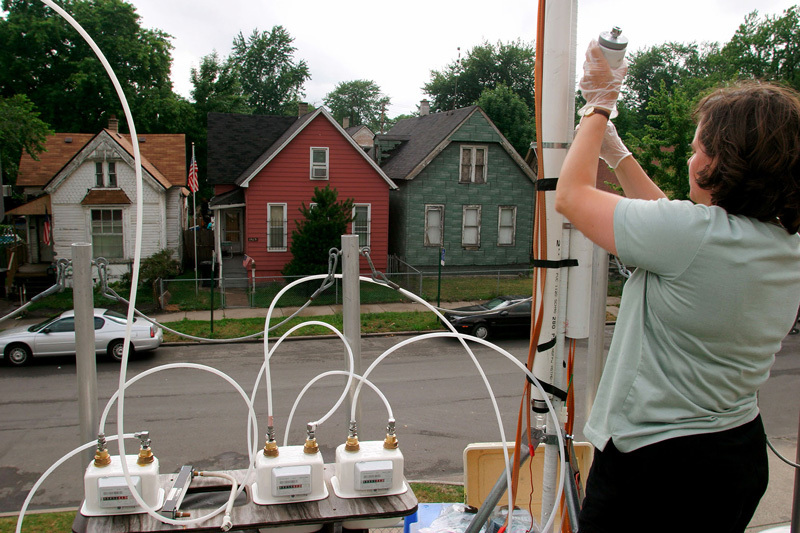
A researcher changes the filter on a mobile air sampler parked near Maybury Elementary School, an area of Detroit with an especially high prevalence of asthma. © Jim West/Alamy

“This area is not well,” she says, pointing to a number of localized health problems. At the head of the list are asthma and cardiovascular disease, both of which have been studied for decades in connection with air levels of particulate matter measured at various points in Detroit and southeastern Michigan.^13^^,^^14^^,^^15^^,^^16^^,^^17^^,^^18^

In the late 1990s, growing awareness of Detroit’s high prevalence of asthma prompted the creation of Community Action Against Asthma (CAAA), a community-based participatory research partnership. Members of CAAA have drawn attention to the links between air pollution and this chronic condition, and have gone into local homes to educate individuals about symptoms and prevention.^19^

Heightened occurrence of asthma in Detroit and other major cities has been attributed to proximity to major roads,^20^ industrial emissions such as sulfur dioxide (SO_2_),^21^ and indoor exposures such as cockroach antigens.^22^ For her part, Anderson is suspicious of piles of black, carbonaceous material known as petroleum coke (“petcoke”) that sat along the river for several months. She acknowledges that petcoke may not necessarily have been any worse for residents’ health than the SO_2_ emissions from nearby steel plants. But she regards the black piles as a greater source of stress for residents, a visible reminder of contamination in their surroundings. “You don’t get that same sense from issues like SO_2_ because you can’t see it,” she explains.

A by-product of oil refining and similar hydrocarbon-cracking operations, petcoke can be used much like coal. For the Marathon Petroleum Corporation, which operates a large refinery near Delray, petcoke is an attractive resale item. After Marathon began processing heavier crude from the oil sands of Alberta in 2012, the refinery’s output of petcoke increased.^23^ Large piles of the material began to appear on the Detroit River waterfront, awaiting shipment to buyers, where they attracted media and political attention.^24^ The mayor of Detroit ordered the piles moved, and by the end of 2013, owner Koch Carbon had relocated the piles to sites outside the state.^25^ In July 2014 the Michigan Department of Environmental Quality denied an application to allow bulk storage of petcoke along the river, citing the inadequacy of proposed measures to control fugitive dust.^26^

## Neighborhood Health

Although the petcoke is gone, many other piles of material—coal, salt, sand, limestone, and more—still line the Detroit River as they have for decades, both fuel for and fruit of the city’s industries. Just off the river, perched between Delray and the southwestern city limits of Detroit, is Zug Island, which has achieved near-mythic status in the pantheon of North America’s most heavily industrialized places. Created in the late 1800s when developers bought 325 acres of land and carved a canal around it, the island became home to a large blast furnace at the beginning of the twentieth century. The steel operations expanded and changed hands over the ensuing decades, becoming what is now the Great Lakes Works facility of United States Steel Corporation.^27^

Zug Island’s environmental notoriety has been fostered by incidents such as a 1998 fire at a Honeywell International tar plant, which was subsequently cited by the Environmental Protection Agency for releasing substantial amounts of carcinogenic benzo[*a*]pyrene and dibenz[*a,h*]anthracene, a probable human carcinogen.^28^ Even the sound of the place has drawn attention, as Canadians living across from Zug Island have blamed it for an intense hum that some mistook for an earthquake.^29^^,^^30^ Travelling southwest down the Detroit River reveals several steel mills, coal-fired power plants, and one of the continent’s largest waste incineration plants.

Even so, comparisons with other cities have placed Detroit somewhere in the middle of the pack with respect to environmental health risks associated with fine particulate air pollution.^16^ Similarly, monitoring between 2001 and 2007 actually showed a decline in the concentrations of most of the air pollutants identified as agents of concern, among them arsenic, nickel, formaldehyde, and benzene.^31^ While improved air-quality control measures may have played a part in this drop, other possible factors include business closures and reduced traffic volumes—results of the economic and social turmoil that has caused the city to lose so many businesses and people.^31^

**Figure:**
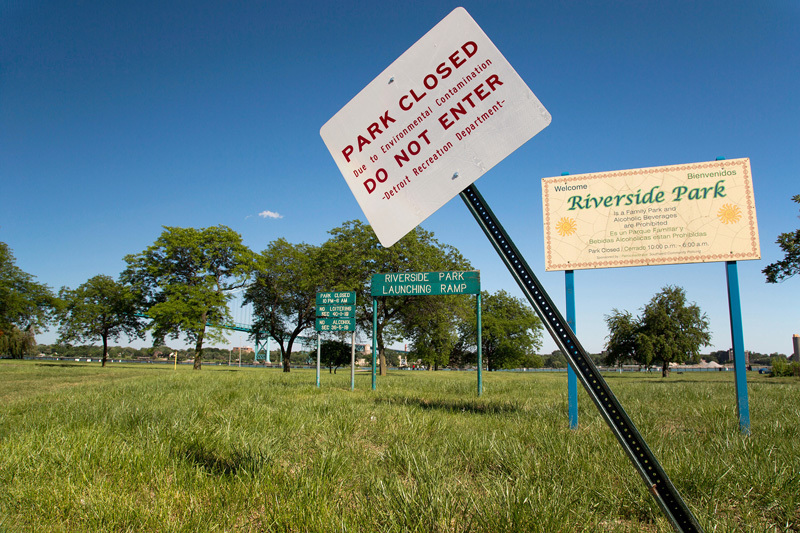
The environmental integrity of city parks is sometimes compromised by nearby industrial activities that cause water or soil contamination. Riverside Park was closed in 2012. © Jim West/Alamy

As residents and researchers continue to assess Detroit’s environmental health, they are beginning to pay more attention to the role of residents’ perceptions in determining quality of life. And besides studying how air pollution in the region affects physical health outcomes such as asthma,^15^^,^^32^^,^^33^ the mental health impacts also have been explored.^34^^,^^35^^,^^36^^,^^37^

From 2008 to 2013, teams led by the University of Michigan’s School of Public Health worked with local volunteers to collect detailed mental and physical health information from 1,500 randomly selected city residents in an initiative called the Detroit Neighborhood Health Study. Sandro Galea, chairman of the Columbia University Department of Epidemiology, was already steeped in this field when he became the principal investigator of the study. “Our group’s research agenda has long been on mental health and how it is produced in urban environments,” he says. “To that end we were interested in highlighting this in the Detroit context.”

In addition to providing tissue samples, study participants also answered questions about elements of psychological well being, including food security, presence of a social network, and exposure to trauma, such as violent experiences or the sudden death of a loved one. The researchers also collected information about the state of each neighborhood’s infrastructure, including the number of abandoned homes and whether sidewalks were maintained. This undertaking has made it possible to create city maps (yet unpublished) outlining levels of post-traumatic stress disorder, depression, and generalized anxiety disorder.

“The duality between mental and physical health is, to my mind, historical and not such a useful paradigm any more,” Galea says. “The two are not separate—they are, rather, both manifestations of physiologic imbalance and are deeply intertwined.”

## Modern Disparities

Galea suggests that Detroit is not unique but instead paradigmatic of communities around the world that depend on heavy industry. “Detroit represents, unfortunately, an interesting case study in urban adversity,” he says. “However, it is important to remember that most people are resilient to such adversity and are thriving, despite the challenges.”

Paul Mohai has had his own close encounters with this adversity and resilience, leaving him all the more interested in the tears and repairs to Detroit’s urban tapestry. His curiosity was originally piqued soon after he joined the University of Michigan School of Natural Resources and Environment in 1987, when he encountered *Toxic Wastes and Race in the United States*, a provocative report by the Commission for Racial Justice that found hazardous waste sites were disproportionately located in areas inhabited by racial and ethnic minorities and the poor.^38^

**Figure:**
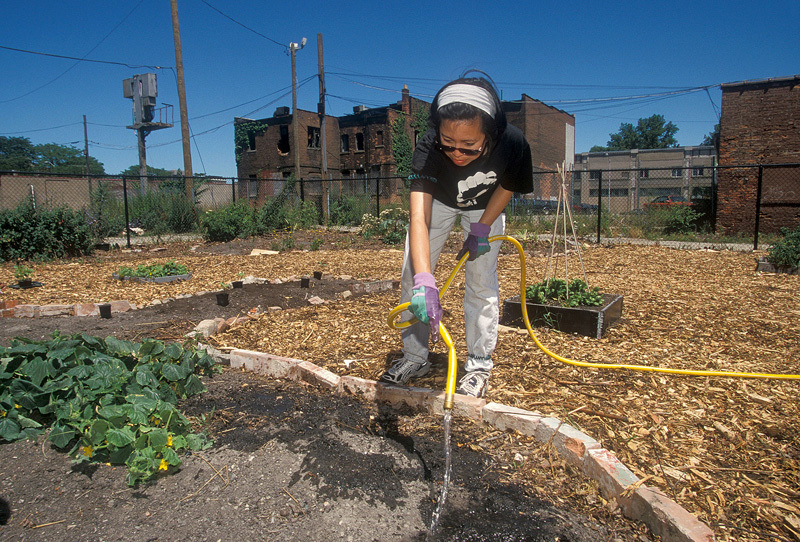
Community gardens are becoming part of the cultural identity in Detroit, where ambitious residents turn vacant lots into urban farms. © Jim West/Alamy

“The report found that the people-of-color percentages in zip codes containing hazardous waste facilities were double that of zip code areas that contained none,” he says. “Furthermore, it found that the people-of-color percentages were tripled in zip code areas containing two or more such facilities. A multivariate statistical analysis also demonstrated that the minority percentage of the zip code area was the best predictor of which zip codes had hazardous waste facilities in them and which did not.”

For Mohai, this finding spawned an ongoing interest with how some groups bear a distinct environmental burden. In the early 1990s he and his colleague Bunyan Bryant, now director of the Environmental Justice Initiative at the University of Michigan, measured the distance of a probability sample of Detroit area residents to nearby hazardous waste sites and polluting industrial facilities. They found that black residents consistently lived closer to such sites than white residents.^39^^,^^40^ They furthermore discovered that Michigan was the state with the greatest racial disparities around hazardous waste facilities, with people of color making up 66% of the residents living within 3 km of such facilities versus 19% of the residents living further away.^41^ Other state- and national-level studies showed similar patterns.^4^^,^^42^^,^^43^

But Mohai says historical context is essential to understanding how some neighborhoods become demographically challenged and environmentally compromised. In one of his statewide studies, Mohai and colleague Robin Saha, an associate professor of environmental studies at the University of Montana, found little evidence to show that facilities constructed in Michigan before 1970 were sited disproportionately in minority and poor communities. It was not until after 1970, they found, that such patterns began to emerge.^4^

**Figure:**
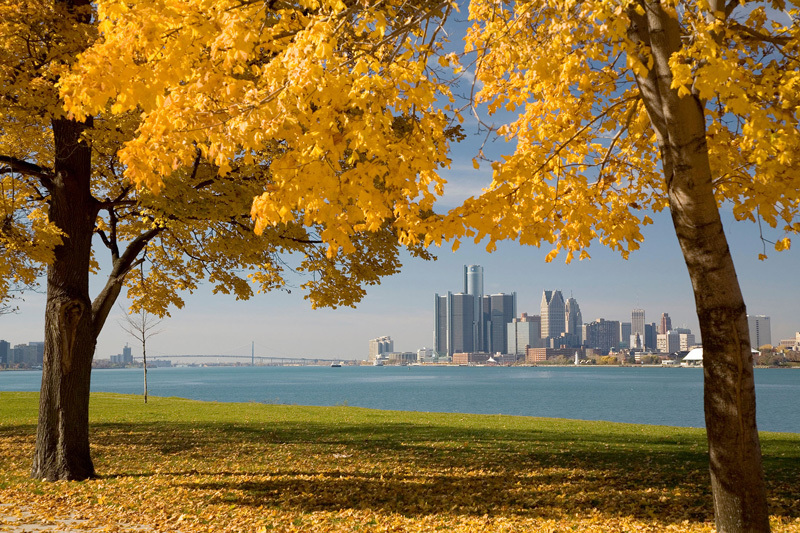
Belle Isle is a popular Detroit park once accessible only by boat. © Jim West/Alamy

**Figure:**
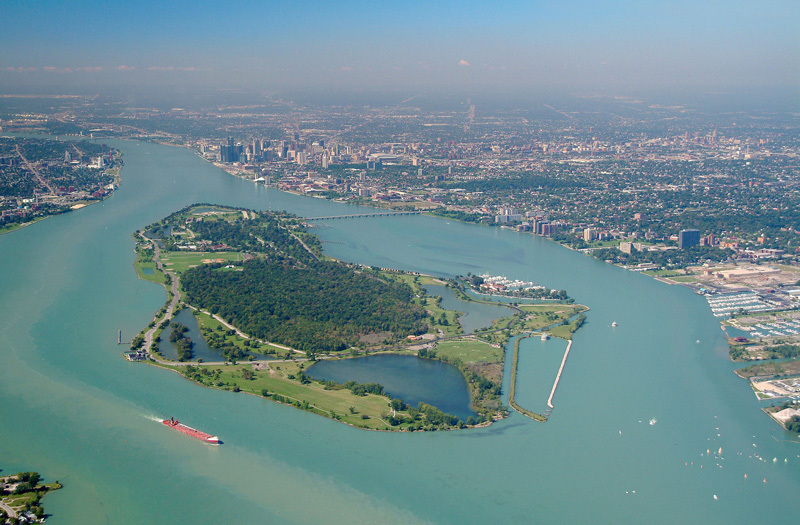
Restorations to the long-neglected Belle Isle facilities aim to attract both people and wildlife. © Marvin Dembinsky Photo Associates/Alamy

Mohai and Saha hypothesize that growing environmental awareness in the United States during the 1960s—which culminated in the first Earth Day demonstrations in the spring of 1970,^44^ the passage of the National Environmental Policy Act (NEPA),^45^ and the creation of the Environmental Protection Agency^46^—led to increasing concerns about the harmful effects of pollution in people’s neighborhoods. These concerns were further heightened by coverage of the Love Canal disaster, in which residents discovered their Niagara Falls neighborhood had been built over a hazardous waste site and the waste was seeping into their homes and backyards.^47^

NIMBYism—“not in my backyard” syndrome—became an increasing response to growing public concerns about environmental contamination, Mohai says. “Unequal resources and political representation and clout put poor and [minority] communities at a disadvantage in trying to resist the siting of hazardous waste and other locally unwanted land uses—LULUs—in their backyards,” he says. “Indeed, we found that the greatest racial and socioeconomic disparities in [new] facility siting occurred in the decade right after the Love Canal story broke in 1979.”

Mohai maintains that siting decisions have not always been made to the disadvantage of poorer people. “At one point we weren’t noticing pollution,” he says. “We weren’t worrying about the danger.” However, he adds, the environmental movement and subsequent research, education, policy making, and awareness have resulted in significant and permanent changes in how many view and approach environmental problems.

## “We Hope for Better Things”

That time before worry is well within living memory for Dolores Leonard, a retired college professor who was born in River Rouge, which abuts Delray. Now approaching 80, she remembers at age 12 asking her father why people placed tarpaulins over their parked cars. Even after learning it was to prevent soot from pitting the paint, she says it didn’t cross her mind until she was a young parent herself to consider what that same air pollution might be doing to people who breathed it. “We took for granted Great Lakes Steel just spewing out whatever it was spewing out,” she says. “And if the sky was red, or it was orange or gold—whatever it was, we just accepted that to be normal. We did not know that it was impacting our health.”

**Figure:**
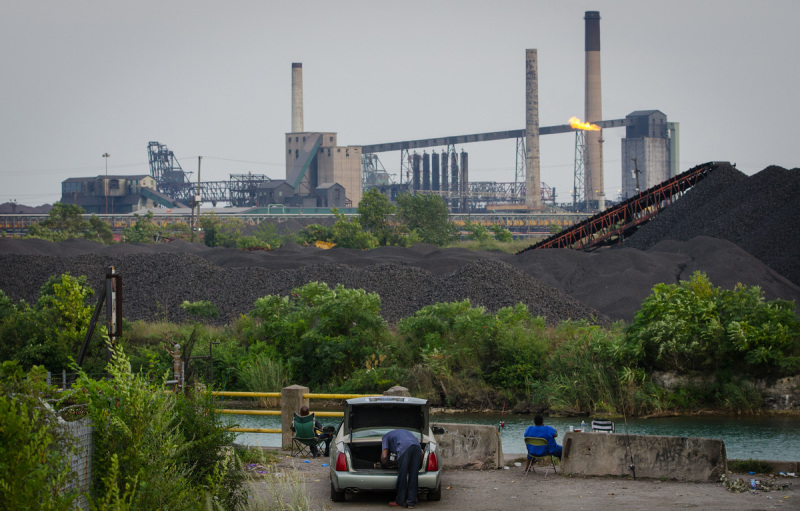
Local fisherman try their luck across from Zug Island, undaunted by the activity at United States Steel Corporation. © James Fassinger

Today we do know, Leonard adds, and that knowledge led her to join the ranks of Detroit’s lively community of environmental health advocates. Their effect on the region has been palpable. The city is becoming known for its thriving urban agriculture movement, as depopulated neighborhoods put empty lots to work as community gardens.^48^ Miles of downtown Detroit River waterfront are being transformed into an extensive paved walkway,^49^ and Belle Isle, a popular park once accessible only by boat, is being renovated after years of neglect, with restorations aimed at enticing both people and wildlife back to the site.^50^

Back near River Rouge and Delray, residential and corporate citizens are attempting to embellish the natural spaces that remain in this heavily industrialized landscape. In the Oakwood Heights neighborhood, neighboring Marathon Petroleum launched a two-stage buyout in 2011 to reshape the area in the wake of a $2.2-billion upgrade and expansion of the refinery. Homeowners were offered a premium price for their homes, starting at $50,000, around 50% more than the average appraised value of local properties. Some 90% of homeowners enrolled in this program, which eventually gave Marathon access to hundreds of vacant lots.^51^ The company subsequently hired the nonprofit Detroit Greenworks, which trains local residents in landscaping and forestry, to begin cleaning up these properties in preparation for more ambitious green-space plans.

One afternoon this fall, Marathon representative Honor Sheard came out to Delray Neighborhood House to discuss ongoing efforts with a group of journalists touring the city. When bluntly asked by one reporter what it means to work for a company that still has such a profound environmental footprint, Sheard was equally blunt in stating that the company is dedicated to improving whatever it can. “I have some land, I have some money,” she told the reporter. “How do I make this site that I can control better? How can I bring something to the community? How can I make the community a little brighter?”

Sheard was articulating the same commitment heard from so many residents, that this is home and leaving is not an option. For Leonard, that commitment is visceral. “Not everyone can leave the community,” she says. “If you have skills, if you have knowledge, it behooves you to bring the others up and help. You can’t be selfish. You have to stay, and you have to help.”
